# Bergamot Polyphenols Improve Dyslipidemia and Pathophysiological Features in a Mouse Model of Non-Alcoholic Fatty Liver Disease

**DOI:** 10.1038/s41598-020-59485-3

**Published:** 2020-02-13

**Authors:** Vincenzo Musolino, Micaela Gliozzi, Federica Scarano, Francesca Bosco, Miriam Scicchitano, Saverio Nucera, Cristina Carresi, Stefano Ruga, Maria Caterina Zito, Jessica Maiuolo, Roberta Macrì, Nicola Amodio, Giada Juli, Pierfrancesco Tassone, Rocco Mollace, Rebecca Caffrey, Jonathon Marioneaux, Ross Walker, James Ehrlich, Ernesto Palma, Carolina Muscoli, Pierre Bedossa, Daniela Salvemini, Vincenzo Mollace, Arun J. Sanyal

**Affiliations:** 1 Institute of Research for Food Safety and Health (IRC-FSH), Department of Health Sciences, University “Magna Graecia” of Catanzaro, Catanzaro, Italy; 2 Nutramed S.c.a.r.l. Complesso Ninì Barbieri, Roccelletta di Borgia, Catanzaro, Italy; 3 Department of Experimental and Clinical Medicine, University “Magna Graecia” of Catanzaro, Catanzaro, Italy; 4 Sanyal biotechnology, 800 E Leigh St, Richmond, VA 23219 USA; 5 Macquarie University Medical School, Sydney, Australia; 6 University of Colorado, Denver, CO USA; 7 Liverpat, Paris, France; 8 Institute of Cellular Medicine, University of Newcastle, Newcastle, UK; 9 Department of Pharmacology and Physiology, Saint Louis University School of Medicine, 1402 South Grand Blvd, St. Louis, MO 63104 USA

**Keywords:** Mouse, Target identification, Metabolic syndrome, Non-alcoholic fatty liver disease, Non-alcoholic steatohepatitis

## Abstract

There is a need for continued drug development for nonalcoholic steatohepatitis (NASH). Bergamot is a plant whose fruit juice is enriched with flavonoids and phenolic compounds which improves dyslipidemia and markers of systemic inflammation in patients with Metabolic Syndrome. The aim of this study was to perform a preclinical “proof of concept” study of Bergamot polyphenolic formulation (BPF99) for the treatment of NASH. A disease reversal study was performed in the diet-induced animal model of NAFLD (DIAMOND). Groups of 8 weeks old mice were randomly assigned to receive chow diet, high fat diet with sugar in drinking water (Western diet- WD). Mice on WD were further randomized to continue on WD gavaged with vehicle or continue on WD with additional gavage of BPF99 (50 mg/kg) after 16 weeks of diet. Mice were euthanized after 11 additional weeks. The primary endpoint was resolution of NASH. Secondary endpoints included changes in individual histological features, body weight, liver enzymes, dyslipidemia, markers of oxidative stress and molecular markers of disease activity and fibrosis. The results showed that BPF99 reduced ALT (mean 71.6 vs 44.6 IU/l, p < 0.01), triglycerides (38.8 vs 28.1 mg/dl, p < 0.05), LDL-C (39.2 vs 23.7 mg/dl, p < 0.001). It significantly improved NASH resolution (p < 0.001) and the SAF scores (p < 0.05) while the NAS improvement approached significance. BPF99 reduced markers of oxidative stress, along with reduced JNK and p38 MAP kinase activity. BPF99 did not reduce the number of mice with fibrosis but improved collagen proportional area (p < 0.04) and procollagen I and III expression. Collectively our results showed that BPF99 resolves NASH and ameliorates key histological and pathophysiological features of NASH along with improvement in ALT and dyslipidemia in the DIAMOND mice.

## Introduction

Nonalcoholic fatty liver disease (NAFLD) is a major cause of liver-related morbidity and mortality for which there are no approved therapies^1^. The clinical-histological spectrum of NAFLD extends from a nonalcoholic fatty liver (NAFL) to nonalcoholic steatohepatitis (NASH)^2^. NASH is a more aggressive phenotype of NAFLD and is more likely to progress to cirrhosis^3,4^. Excess liver-related mortality from NASH is mainly due to progression to cirrhosis and development of hepatocellular cancer. Evidence exists that, in the early steps of NAFLD, accumulation of fat in liver tissue occurs as a consequence of the imbalance between overconsumption of high-fat diet and increased *de novo* lipogenesis^5^, on one hand, and decreased lipid disposal, mainly through free fatty acid (FFA) oxidation^6^, on the other. The cellular stress produced either directly by lipotoxicity or the cellular response to metabolic overload drives cell-death and inflammatory signaling via activation of the innate immune system^7^. In this scenario, some pathways including oxidative stress and the unfolded protein response^8,9^ contribute to the development of chronic inflammation, inducing a fibrogenic response and progressive fibrosis leading to cirrhosis.

Thus, a better understanding of the steps involved in regulating lipid accumulation, inflammation and oxidative stress might provide a new therapeutic strategy for NAFLD prevention and treatment. Many therapies are directed towards targets that are expected to reduce disease activity e.g. pioglitazone and glucagon like peptide-1receptor agonists and thus eventually translate into decreased disease progression whereas others directly target fibrogenic pathways. The current regulatory pathway both in Europe and in the United States requires demonstration of decreased disease activity, measured by frequency of resolution of steatohepatitis or decrease in disease activity scores, without any independent adverse impact on fibrosis stage in the short term and/or improvement in fibrosis without worsening of disease activity^10^. In the long-term, drugs reducing disease activity should reduce progression to cirrhosis. Alongside with the pharmacological interventions suggested in the last decades to approach NAFLD/NASH, lifestyle modifications, consisting of exercise, low-fat and fruit and vegetable-based diet, are the cornerstone of therapy for fat-related liver dysfunction^11^.

In particular, it has been shown that polyphenols, which are found ubiquitously in plants, and their regular consumption are associated with a reduction in the risk of a number of metabolic diseases, including NAFLD^12^.

Bergamot (*Citrus bergamia Risso et Poiteau)* is an endemic plant growing in Calabria (Southern Italy), which has a particular composition and high content of glycosylated flavanones and flavones^13^.

Bergamot Polyphenol Fraction (BPF99) is a juice extract containing 47% of flavonoids (naringine, neohesperidine, neoeriocitrine, brutieridine and melitidine) as well as traces of sugars, salts and other natural compounds^14^. A particular feature of certain polyphenols, abundant in BPF99, is the potential protective cellular activity as demonstrated by several *in vitro* experiments. Particularly, naringine is known to exert a protective action on oxidative damage in tert-butyl hydroperoxide-induced HepG2 injury^15^ and in rat hepatocytes against toxin-induced overphosphorylation and disruption of the keratin cytoskeletal network, as well as against toxin-induced apoptotic cell death^16^; in addition it has shown anti-inflammatory, antioxidant and antiapoptotic activities on H9C2 cells^17,18^ and on RAW 264.7 macrophages^19^. Then, neohesperidin regulates lipid metabolism via FGF21 and AMPK/SIRT1/PGC-1α signaling axis^20^. A peculiarity of bruteridin and melitidin is the ability to bind the catalytic site of HMG-CoA reductase and inhibit cholesterol synthesis by replacing its endogenous substrate HMG-CoA^21^. Recently, it has ben reported that BPF99 phytocomplex was able to modulate autophagy in HepG2 cells in a time- and dose-dependent fashion and the effect was amplified in cells loaded with palmitic acid^22^. Lastly, it has been shown that an analogue bergamot whole-fruit powder extract exerts antioxidant activity, *in vitro*, showing a selective inhibition against pathogenic strains and a growth stimulation effect on some beneficial gut bacteria. Moreover, the *Citrus* extract, rich in healthful phytochemical compounds, was able to protect human microvascular endothelial cells from LPS-induced activation and dysfunction, reducing the endoplasmic reticulum stress^23^.

The pleiotropic effects of these flavonoids contained in BPF formulation can justify the anti-oxidative and anti-inflammatory properties observed in patients suffering from metabolic syndrome^24,25^.

In this population, bergamot polyphenols reduce LDL-cholesterol (LDL-C), and increases HDL-cholesterol (HDL-C) as well as markers of systemic inflammation linked to cardiovascular outcomes such as the hs-CRP^26–28^. Recently, it has also been shown to improve the results of the Steato-Test in this population^27^. Altogether, these discoveries provide a rationale to further evaluate its potential impact on underlying NASH.

Prior to engaging in human trials, the objective of this study was to provide preclinical proof of concept that BPF99 can improve the histological features of NASH in a preclinical model of NASH that has been validated to resemble most features of human disease. The diet-induced animal model of NAFLD (DIAMOND) is one such model; it is an isogenic cross of a C57Bl6/J and s129svlm6 mouse which reproducibly develops obesity, adipose tissue inflammation, hypoadiponectinemia, increased IL-6 and TNF-α, insulin resistance, dyslipidemia and NAFL which progresses to NASH with histology that resembles human NASH and progresses to bridging fibrosis and early nodule formation^29^. The key cellular signalling pathways relevant for human NASH are also activated in this model and the evolution of the transcriptomic changes with disease evolution are concordant with human NASH^30^. Further, the lipidome of the DIAMOND mouse is also similar to human NASH^31^. We therefore tested the effect of BPF99 on the histological severity of NASH in the DIAMOND mouse. Secondary analysis of the effects of BPF99 on key pathways related to lipogenesis, cell stress, apoptosis, inflammation and fibrosis were also assessed.

## Materials and Methods

### Animals

Male DIAMOND (diet-induced animal model of non-alcoholic fatty liver disease) mice were purchased from Sanyal Biotechnology (Virginia Beach, VA, USA) kept under standard laboratory conditions in a specific‐pathogen-free animal facility and maintained at 22 ± 2 °C with alternating 12 h light–dark cycle. All the experimental procedures were performed according to protocols approved by the Animal Care of University Magna Graecia of Catanzaro, in accordance with the European Commission guidelines (Directive 2010/63/EU) for the animals used for scientific purposes. The protocol was further reviewed and approved by the IACUC at Eastern Virginia Medical School AALAC-certified animal research facility where the animal studies were performed. Two mice were maintained in each cage and mouse handling and care was provided in accordance with recommendations from the mouse metabolic phenotyping facility.

### Bergamot polyphenolic formulation (BPF99) preparation and dilution

BPF99-Bergacyn, as prepared and characterized for polyphenols content^24^, was provided by Herbal and Antioxidant Derivatives (H&AD), (Bianco, Reggio Calabria, Italy). Water was used as a vehicle to dilute BPF99 to a final concentration of 0.015 mg/μl. Animals were gavaged once per day with a calculated volume of solution contained a dose of 50 mg/kg BPF99. Gavage was given without anesthesia.

### Study design

**Figure 1:**
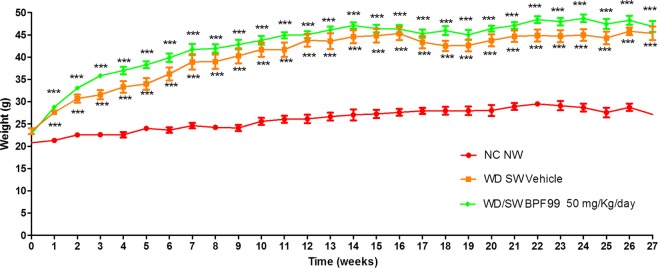
Effect of BPF99 on body change over time. Animals were fed a normal chow diet and tap water (NC NW) or high fructose/glucose, high fat Western Diet (WD SW) for up to 27 weeks. WD SW animals were treated with vehicle (n = 10, orange line) or 50 mg/kg/day BPF99 (n = 10, green line), via gavage once daily over a period of 11 weeks, starting from week 16. NC NW mice (n = 5, red line) did not receive any treatment. Data are expressed as the mean ± SEM. ***p < 0.001 vs NC NW.

Mice of 8–12 weeks of age and weight of 22.6 ± 0.4 g were fed *ad libitum* a normal chow diet (NC, Harlan TD.2019) and tap water (NW) or a high fat/high carbohydrate diet (Western diet, WD, Harlan TD.88137) and with a high fructose-glucose water solution (SW, 23.1 g/L d- fructose + 18.9 g/L d-glucose) for up to 27 weeks. The choice of diet was based on previously published studies^32^. WD SW animals were treated with vehicle (n = 10) or 50 mg/kg/day BPF99 (n = 10), via gavage once daily over a period of 11 weeks, starting from week 16. NC NW mice (n = 5) did not receive any treatment (Fig. 1).

One day before starting diet regimen, baseline body weight was assessed. Animals were weighed once a week till the end of the study. The day of the sacrifice, animals were exposed to inhaled isoflurane prior to being euthanized. Euthanasia was performed by cervical dislocation. The entire liver was removed from the abdominal cavity and weighed. The liver was sectioned in a sagittal plane and placed in containers of 10% formalin, for later histologic processing and analysis, or into separate cryotubes and immediately frozen in liquid nitrogen for further analysis.

### Histological analysis

Liver histology was assessed from paraffin-embedded tissue sections stained with hematoxylin and eosin or picrosirius red. Histology was reviewed using the NASH-Clinical Research Network (CRN) criteria and fatty liver inhibition of progression (FLIP) algorithm by an expert liver pathologist (Dr. Pierre Bedossa), blinded to the treatment group. Given the similarity of the histological expression of disease in the DIAMOND mice vis-à-vis human NAFLD, the presence of steatohepatitis was diagnosed as described previously^29^.

For each liver slide, the main histological lesions were assessed using the Steatosis-Activity-Fibrosis (SAF) score system^33,34^. Steatosis was graded on a scale of 0 to 3 (0: < 5%; 1: 5–33%; 2: 34–66%; 3: >67%).

Grade of activity (0–2) is given by the sum between the presence of balloning and inflammation. Ballooning hepatocytes was graded as 0 (none), 1 (when few hepatocytes presented a rounded shaped, reticulated and pale cytoplasm, but with normal dimensions), and 2 (when there is a cluster of prominent balooning hepatocytes).

The presence of inflammatory foci within the lobule or within the sinusoids, was graded as 0 (none), 1 (<2 foci per 20x field), 2 (2–4 foci per 20x field), and 3 (>4 foci per 20x field).

The NAFLD activity score (NAS) was calculated by addition of grades of steatosis, inflammation and ballooning^2^. Detection of collagen fibers was performed by picrosirius red staining and fibrosis stage was scored according to the NASH-CRN system^2^. Fibrosis was also quantified by morphometry and expressed as collagen proportionate area (CPA)^35^.

Then, FLIP algorithm was used for classification of liver slides in steatosis-without-NASH and steatosis-with-NASH, in according to the SAF score^34^.

### Assessment and quantification of protein nitrotyrosine adducts in the liver

Oxidative damage in the liver was assessed by immunohistochemical staining for 3-nitrotyrosine (3-NT), a biomarker of oxidative damage mediated by peroxynitrite^36^. Formalin-fixed, paraffin-embedded sections (5 µm thick) were deparaffinized by exposure to xylene and graded alcohols and then washed in water. Epitopes were retrieved by heating the slides to 98 °C for 30 minutes in citrate buffer (10 mM Sodium Citrate, 0.05% Tween 20, pH 6.0). Endogenous peroxidase activity was suppressed by immersion of the slides in a peroxidase suppressor (#35000, Thermo Scientific, Waltham, Massachusetts, USA) for 30 minutes and then washed in a washing buffer (25 mM Tris, 0.15 M NaCl, 0.05% Tween 20, pH 7.2). Slides were blocked in a blocking buffer (#1859332, Thermo Scientific, Waltham, Massachusetts, USA) for 30 minutes. 3-nitrotyrosine was stained by exposure to anti-nitrotyrosine antibody (#06-284, Millipore, Burlington, Massachusetts, USA; 1 mg/mL) in the blocking buffer (#1859332, Thermo Scientific, Waltham, Massachusetts, USA) for 120 minutes at room temperature. After the incubation period, each slide was washed 2 times with a washing buffer (25 mM Tris, 0.15 M NaCl, 0.05% Tween 20, pH 7.2) and incubated with a secondary antibody horseradish peroxidase (HRP)-conjugate (#31460, anti-Rabbit, Thermo Scientific, Waltham, Massachusetts, USA) for 1 h at room temperature, followed by 3,3-diaminobenzidine (#1856090, Thermo Scientific, Waltham, Massachusetts, USA). Hematoxylin (#1859352, Thermo Scientific, Waltham, Massachusetts, USA) was used for nuclear counterstaining. Positive staining was visualized by intense brown staining. In each case, a negative control (without primary antibody) was used. Images of liver tissue immunostained for 3-NT were captured using an Olympus microscope (BX53) and high-resolution Olympus digital image camera (XC50). Cellsens Dimension software was used to acquire the images. The intensity of 3-NT staining was assessed using ImageJ (NIH) as described^37^. Positive brown staining was registered between 30 and 115 intensity units while the background and nuclear staining were greater than 160 intensity units. A total of 4 images (40X magnification) from each slide were analyzed for the percent of pixels within the 30–115 intensity unit as measure of immunoreactivity.

### Glucose and insulin tolerance tests

To perform glucose tolerance test (GTT) and insulin tolerance test (ITT), a small blood volume was collected simply puncturing the tail vein with a small gauge needle. Blood glucose levels were measured using a glucometer (MultiCare In, Biochemical Systems International, Arezzo, Italy).

For the GTT, mice were fasted overnight and baseline blood glucose levels were measured. Glucose (1 mg/g body weight) in sterile saline solution was injected intraperitoneally and blood glucose levels were measured 10, 20, 30, 60, 90 and 120 minutes after glucose injection. ITT was performed, three days later, on the same animals used for the GTT. Mice were fasted for 4 h and intraperitoneally injected with 0.75 U/kg body weight regular insulin (Actrapid, Novo Nordisk, Farmaceutici, Rome, Italy). Blood glucose levels were measured at the baseline and 15, 30, 45, 60 and 75 min after insulin injection.

### Blood collection and analysis of blood based endpoints

Blood was collected via cardiac puncture from the heart of euthanized mice. A volume of around 700 μl of whole blood was collected into untreated Eppendorf tubes and centrifuged for 15 min at 1,500 g at 4 °C. The serum was then collected and stored at −80 °C for later analysis.

Alanine aminotransferase (ALT), aspartate aminotransferase (AST), glucose (GLU), total cholesterol (CHOL), low-density lipoproteins (LDL-C) and triglycerides were measured in serum samples, using an automatic chemistry analizer, XL-640 (Erba Mannheim) and the following Erba liquid stable reagents: glucose (GLU 440, XSYS0012), total cholesterol (CHOL 440, XSYS0009), low-density lipoproteins (LDL C 80, XSYS0044), alanine aminotransferase (ALT/GPT 330, XSYS0017), aspartate aminotransferase (AST/GOT 330, XSYS0016) and triglycerides (TG 440, XSYS0041).

The index of the oxidative stress was evaluated by assessing serum lipid peroxidation product malondialdehyde (MDA) using a lipid peroxidase assay kit (Sigma-Aldrich, Saint Louis, USA) according to the manufacturer’s protocol. Briefly, serum sample is first treated with trichloroacetic acid (TCA) for protein precipitation and then treated with thiobarbituric acid. The mixture is heated for 10 minutes in boiling water bath. One molecule of MDA reacts with two molecules of thiobarbituric acid. The resulting chromogen is centrifuged and intensity of colour developed in supernatant is measured colourimetrically at 530 nm.

Serum total antioxidant status (TAS) was measured spectrophotometrically using kit manufactured by Randox (NX 2332) on a Randox RX Monza (Randox Laboratories Ltd, Crumlin, UK). Briefly, the principle of the antioxidant assay is the formation of a ferryl myoglobin radical from metmyoglobin and hydrogen peroxide, which oxidizes the ABTS (2,2′-azino-bis (3-ethylbenzthiazoline-6-sulfonic acid) to produce a radical cation, ABTS∙+, a soluble chromogen that is green in color and can be determined spectrophotometrically at 600 nm. Antioxidants in the added serum, suppress the production of the radical cation in a concentration dependent manner and the color intensity decreases proportionally.

### Extraction and HPLC analysis of flavonoid compounds in serum samples

Processed serum was analyzed by RP-HPLC analysis on a Thermo Scientific Ultimate Dionex 3000 UHPLC equipped with an Hypersil gold column (C18) (dim.mm 250 × 4.6, particle size 5 μm), injections loop 20 μl, flow rate 1 ml/min and a mobile phase consisted of the following solvents: A (H_2_O + TFA 0.1%), B (methanol). Absorbance was obtained at the wavelength of 230 nm, 254 nm and 280 nm. The instrumentation performance, chromatograms, and initial data processing were carried out with using Thermo Scientific Chromeleon Chromatography Data System (CDS) software. (See *Supplementary Materials and methods*).

### Protein extraction and SDS-PAGE Western Blot analysis

Tissue lysates were prepared as previously described^38,39^. Briefly, 200 mg of liver were homogenized in 300 μL ice-cold lysis buffer (100 mM Tris pH 7,6; 300 mM KCl; 0,1% Triton X 100; 10 μL/mL freshly added protease and phosphatase inhibitor cocktails), centrifuged at 20.000 g for 20 min at 4 °C and supernatant was collected. A total of 20 μL of the supernatant was used to determine the total protein concentration by Bradford assay (Quick Start Bradford 1X Dye reagent, Bio-Rad #500-0205, Hercules, CA, USA) using bovine serum albumin (Quick Start Bovine Serum Albumin Standard, Bio-Rad #500-0206, Hercules, California, USA) as a standard. Proteins were heat-denatured for 5 min at 95 °C in sample loading buffer (500 mM Tris/HCl, pH 6.8; 30% glycerol; 10% sodium dodecyl sulfate; 5% β-mercaptoethanol; and 0,024% bromophenol blue), and 20 μg of protein lysate were resolved by sodium dodecyl sulfate polyacrylamide gel electrophoresis and transferred to nitrocellulose membranes (Amersham Protan 0,2 µm NC 10600001, Little Chalfont, United Kingdom). Membranes were then blocked with Tris/HCl (pH 7.6) containing 0.1% Tween 20 and 5% BSA for 1 h and incubated overnight at 4 °C with shaking with the following primary antibodies: anti-NFkB (Cell Signaling #8242, Danvers, MA, USA); anti-phospho SAPK/JNK (Cell Signaling #9251, Danvers, MA, USA); anti-SAPK/JNK (Cell Signaling #9252, Danvers, MA, USA); anti-phospho acetyl-CoA Carboxylase (Cell Signaling # 11818, Danvers, MA, USA); anti-acetyl-CoA Carboxylase (Cell Signaling #3662, Danvers, MA, USA); anti-phospho AMPKα (Cell Signaling #2531, Danvers, MA, USA); anti-AMPKα (Cell Signaling #2532, Danvers, MA, USA); anti-phospho MAPK/p38 (Cell Signaling #4511, Danvers, MA, USA); anti-MAPK/p38 (Cell Signaling #9212, Danvers, MA, USA); anti-poly (ADP-ribose) polymerase (Cell Signaling #9532, Danvers, MA, USA); anti-Caspase (Cell Signaling #9665, Danvers, MA, USA); anti-cleaved Caspase-3 (Cell Signaling #9661); anti Pro-Collagen III N-terminal propeptide (PIIINP, Mybiosource #MBS2090489, San Diego, CA, USA); anti Pro-Collagen type I (COL1A1, Merck Millipore, #ABT257, MA, USA); anti-GAPDH (Abcam ab181602, Cambridge, United Kingdom); anti-Tubulin (Abcam ab6046, Cambridge, United Kingdom). Membranes were then washed in Tris-buffered saline (TBS, pH 7,6) with 0,1% Tween-20 and incubated with horseradish peroxidase-conjugated secondary antibodies (anti-rabbit antibody Pierce #31460 or anti-mouse antibody Pierce #31430, Invitrogen, Carlsbad, CA, USA) for 1 hour at RT with shaking. Bound antibody was visualized using the chemiluminescent kit (ECL WB Detection, GE Healthcare RPN210601819, Little Chalfont, United Kingdom), immunoblots scanning and analyses were performed using an imaging system (UVITEC Imaging Systems, Cambridge, United Kingdom). Quantification of the bands was performed using the ImageJ Software (NIH, Bethesda, MD, USA).

### Sample size estimation

After 24 weeks of diet, 80–100% of mice demonstrate steatohepatitis^29^. Assuming a decrease of the proportion of mice demonstrating steatohepatitis to 20%, a sample size of 10 mice in each group was estimated to provide a power of 0.8 with a p value fixed at 0.05.

### Statistical analysis

Data were analysed with GraphPad PRISM 6.0 (GraphPad Software, Inc., La Jolla, CA, USA). Results are shown as mean ± SEM. Inter-group comparisons were made using analysis of variance (ANOVA) with *post hoc* Bonferroni correction for multiple comparisons as appropriate for normally distributed variables. A *p* value of <0.05 was considered significant.

### Endpoints

The primary endpoint was the proportion of mice with steatohepatitis at the end of the study. The presence of steatohepatitis was aligned with recent case definitions from the liver forum^33^ and absence of steatohepatitis was defined by a ballooning score of 0 along with minimal (grade1) or no lobular inflammation. Additional analyses included comparison of the severity scores for the individual components of NAFLD and comparison of the composite NAFLD activity score (NAS) and Steatosis-Activity-Fibrosis (SAF) scores as well as the collagen proportional area. Other endpoints of interest included body weight, liver weight, measures of glucose tolerance, liver enzymes and functions, and lipids In additional specific markers related to disease pathophysiology were measured including: (1) Metabolic-acyl CoA carboxylase (ACC), AMP kinase and their phosphorylated state; (2) Cell stress and apoptosis: PARP, caspase 3; (3) inflammation: MAP kinases, JNK, NFκB, and (4) Fibrosis-collagen 1 and 3. These markers were measured by Western blots.

## Results

A total of 25 mice was randomly  divided to receive chow diet, Western diet with *ad libitum* sugar at 8 weeks of age from the same birth cohort of mice. Twenty mice on a Western diet with *ad libitum* administration of sugar water were further randomly assigned to receive BPF99 (n = 10) or vehicle, i.e. water, (n = 10) administered by daily gavage from week 16–27 after initiation of Western diet. The control group (n = 5) were fed a standard chow diet and did not receive treatment. All mice tolerated treatment and there were no deaths from study initiation to end of study. BPF99 most abundant flavonoids were identified in serum sample (*Supplementary results*).

### Effect of BPF99 on body weight

Body weight of mice fed a WD SW diet and treated with vehicle, increased significantly, compared to normal chow-fed controls (Fig. 1, orange line; p < 0.001 vs NC NW). Treatment with 50 mg/kg/d BPF99 in mice fed a WD SW diet did not result in body weight loss compared to the vehicle-treated groups (Fig. 1, green line). The liver weight increased significantly in WD SW-fed positive control mice compared to negative controls and was not affected significantly by BPF99 administration.

### BPF99 improved glucose tolerance and insulin resistance

**Figure 2:**
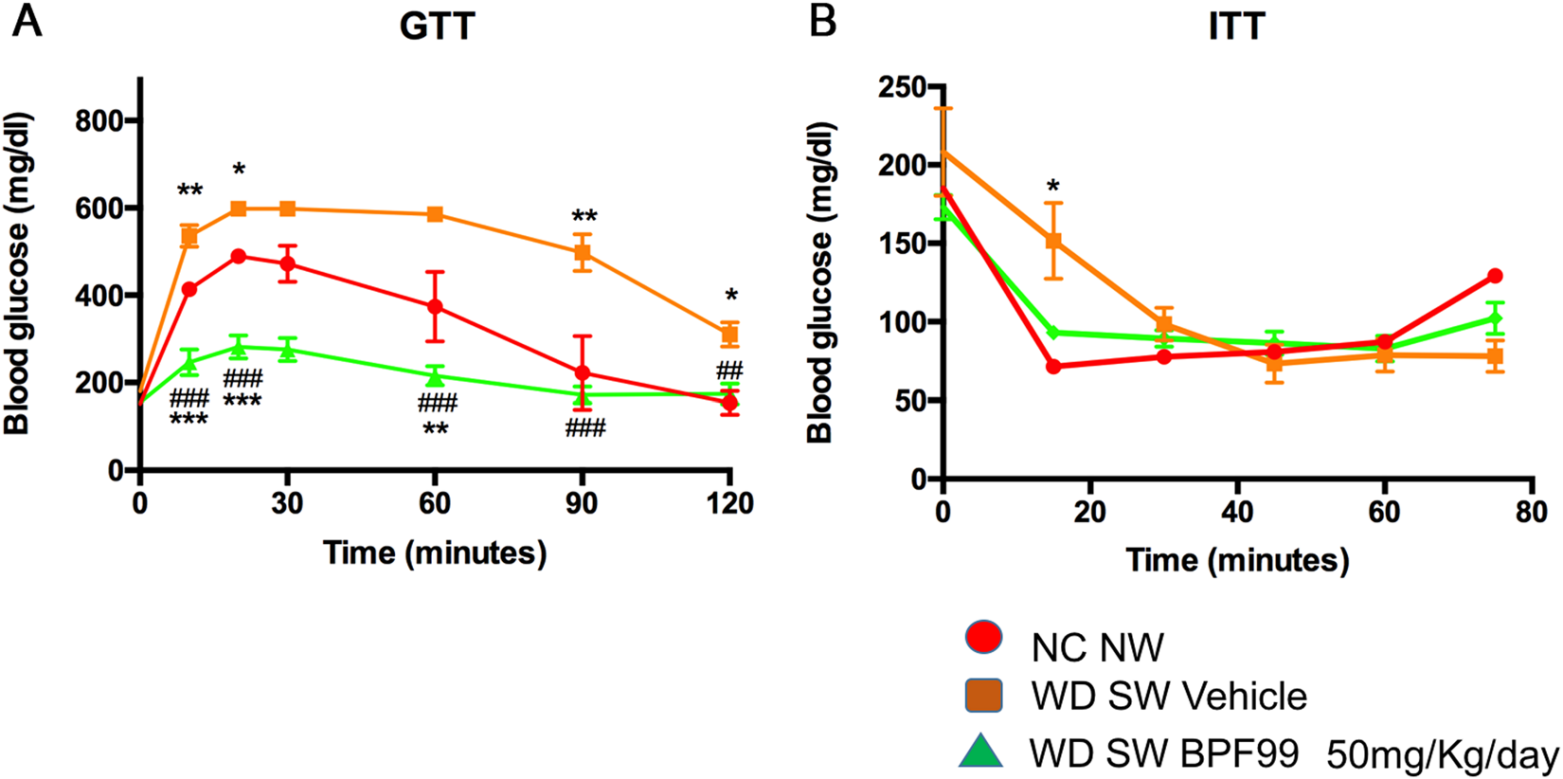
Glucose tolerance test (GTT) and insulin tolerance test (ITT). (**A**) Glucose levels were higher, following intraperitoneal glucose administration, in mice fed a WD SW diet treated with vehicle, compared to NC NW-fed mice. Treatment with 50 mg/kg/d BPF99 significantly enhanced the ability to clear the exogenous glucose in WD SW-fed mice. (**B**) The response to insulin to dispose glucose, was worsened in WD SW-fed mice treated with vehicle compared to CD NW-fed mice. 50 mg/kg/d BPF99 improved insulin resistence in WD SW-fed mice. NC NW (red line); WD SW Vehicle (orange line); WD SW BPF99 (green line). Data are expressed as the mean ± SEM. NC NW AUC = 39419 ± 5000, WD SW Vehicle AUC = 61341 ± 2355, WD SW BPF99 50 mg/Kg/day AUC = 25812 ± 1949; WD SW Vehicle AUC <0.001 vs NC NW AUC. WD SW BPF99 50 mg/Kg/day AUC <0.001 vs WD SW Vehicle. Data are expressed as the mean ± SEM. *p < 0.05 vs NC NW; **p < 0.01 vs NC NW; ***p < 0.001 vs NC NW; ^#^p < 0.05 vs WD SW; ^##^p < 0.01 vs WD SW; ^###^p < 0.001 vs WD SW.

Following glucose administration during a GTT, mice on WD SW had significantly elevated circulating glucose levels with both higher peak glucose and overall glucose area under the curve (AUC) (Fig. 2A). BPF99 administration was associated with a significant improvement in glycemia during the GTT compared to positive controls on a WD SW (p < 0.001 for glucose AUC) as well as mice on chow diet (P < 0.001 for AUC). These indicate that BPF99 improves glucose disposal following a glucose load.

To test the effects of BPF99 on systemic insulin resistance, a key pathophysiological driver of NAFLD, an ITT was performed. Chow diet fed control mice decreased their glucose levels as expected following the insulin challenge. Mice of WD SW had an early significant impairment in insulin sensitivity with higher glucose levels than mice on chow diet after a similar insulin dose; however, over time, they were able to respond and bring their glucose levels down. Administration of BPF99 reversed the early impairment of insulin-mediated hypoglycemia and returned the curve to the pattern seen in negative controls fed a chow diet only (Fig. 2B).

### BPF99 improved liver enzymes and dyslipidemia

Compared to mice maintained on chow diet, mice receiving a WD SW diet had significantly increased AST and ALT levels (p < 0.001 for both) (Table 1). Further, treatment with BPF99 reduced ALT (p < 0.05) compared to mice on WD SW diet without a significant effect on AST levels. WD SW diet also induced an increase in circulating triglycerides, total cholesterol and LDL-cholesterol in the fasted state. As noted with ALT, BPF99 had a significant (p < 0.05 for all compared to WD SW diet alone) beneficial effect on circulating triglycerides, total cholesterol and LDL-cholesterol despite ongoing consumption of a WD SW diet. Although fasting hyperglycemia induced by WD SW diet was decreased by BPF99, this did not reach statistical significance.

**Table 1:** Serum biochemical parameters.

Test	NC NW	WD SW VEHICLE	WD SW BPF 50 mg/Kg/day	NC NW vs WD SW VEHICLE	P Value
					WD SW Vehicle vs WD SW BPF 99 50 mg/Kg/day
Alanine Aminotransferase (U/Liter)	34,58 ± 3,14	71,63 ± 11,59	44,63 ± 6,57	<0,01	<0,05
Aspartate Aminotransferase (U/Liter)	87,33 ± 8,87	121,9 ± 9,87	112,9 ± 14,25	<0,05	0,97
Triglycerides (mg/dL)	20 ± 2,34	38,8 ± 5,38	24,14 ± 3,6	<0,05	<0,05
Total Cholesterol (mg/dL)	105 ± 9,04	242,4 ± 19,50	166,3 ± 10,42	<0,001	<0,01
Low-Density Lipoprotein (mg/dL)	13,57 ± 1,1	39,25 ± 5,31	23,71 ± 2,6	<0,001	<0,05
Fasting glucose (mg/dL)	153 ± 10,91	220,6 ± 22,78	179,6 ± 16,9	<0,05	0,18

Biochemical values were measured at the end of the study in serum of NC NW (n = 5), WD SW Vehicle (n = 10) and WD SW BPF99 50 mg/Kg/d (n = 10) mice. Data are expressed as the mean ± SEM. p values < 0.01, <0.05, <0.001 are considered significant.

### BPF99 significantly increased NASH resolution (primary endpoint)

**Figure 3:**
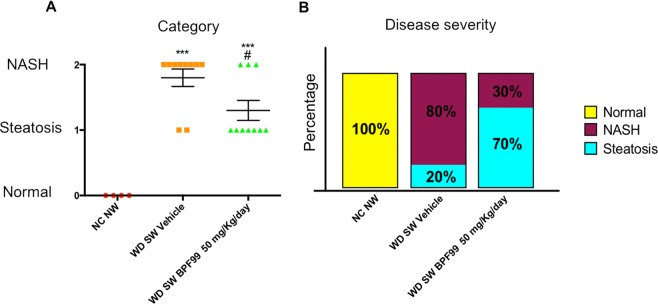
Effect of BPF99 on NASH development. (**A**) Category. None of the negative controls developed steatohepatitis. In contrast, 8 out of 10 mice on a high fat diet with *ad libitum* sugar water administration had steatohepatitis. In the BPF99 group, 3 out of 10 mice developed steatohepatitis. (**B**) Disease severity expressed in percentage. The 80% of mice on Western diet and sugar water had steatohepatitis. Treatment with BPF99 50 mg/Kg/day significantly reduced the number of mice with steatohepatitis. In this arm, only 30% of the animals developed NASH. None of the negative controls developed NASH at the end of study. NC NW (n = 4, red circle); WD SW (n = 10, orange square); WD SW BPF99 50 mg/Kg/day (n = 10, green triangle). Data are expressed as the mean ± SEM. ***p < 0.001 vs NC NW; ^#^p < 0.05 vs WD SW.

None of the negative controls i.e. on chow diet developed steatohepatitis at the end of study. In contrast, 8 out of 10 mice on a high fat diet with *ad libitum* sugar water administration had steatohepatitis using the a priori criteria established (p < 0.001 vs negative controls, Fig. 3A,B). BPF99 significantly reduced the number of mice with steatohepatitis at end of study despite similar diet and weight with only 3 out of 10 mice having steatohepatitis (p < 0.02 by chi square, Fig. 3A,B). All of these mice had NAFL and none of the mice had total resolution of NAFLD.

### BPF99 improved disease activity scores

**Figure 4:**
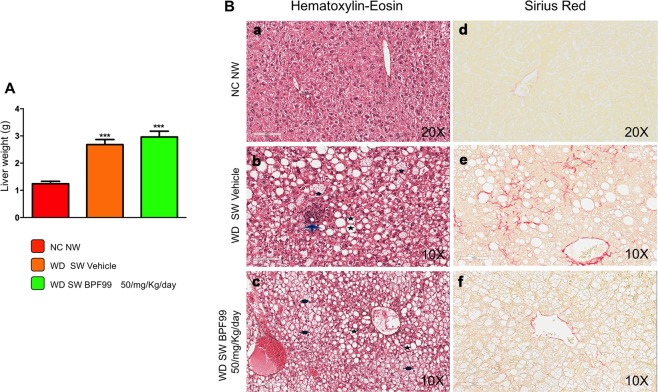
Effect of BPF99 on liver weight and histological features associated with NASH. (**A**) High fat Western Diet was associated with a significant increased in terminal liver weight compared to CD NW-fed animals. 50 mg/kg/d BPF99 did not reduced liver weight in NASH mice. (**B**) Liver histology showed no evidence of steatosis, NASH or sinusoidal fibrosis in mice fed a chow diet and tap water (NC NW, a,d). Steatosis, hepatocyte ballooning, inflammatory foci and clear sign of sinusoidal fibrosis were observed in WD SW-fed mice treated with vehicle (b,e). No inflammation or sinusoidal fibrosis was observed in WD SW-fed mice treated with 50 mg/kg/d BPF99. Representative liver sections stained with hematoxylin-eosin (H&E) (a–c) or picrosirius red (d–f) are shown. Original magnification, x10–x20. (**C**) NC NW (n = 5, red bar or circle); WD SW (n = 10, orange bar or square); WD SW BPF99 50 mg/Kg/day (n = 10, green bar or triangle). Data are expressed as the mean ± SEM. *p < 0.05, **p < 0.01, ***p < 0.001 vs NC NW; ^#^p < 0.05 vs WD SW. Grey star: macrovesicular steatosis; Black arrow: hepatocyte ballooning; Grey arrow: inflammatory foci.

WD SW fed positive control mice had a mean steatosis grade of 2.9 ± 0.1, with 81 ± 3.4% cells with steatosis. BPF99 did not significantly decrease the overall steatosis grade or the portion of steatotic cells. BPF99 also reduced both ballooning grade (p = 0.07) and the proportion of mice with any hepatocellular ballooning. Together, these were reflected in a significant decrease in the SAF activity score (WD SW BPF99 vs WD SW vehicle, p < 0.05, Table 2) while the decrease in NAS approached significance (WD SW BPF99 vs WD SW vehicle, p = 0.054, Table 2). These differences were driven by a lower impact on steatosis grade which is part of the NAS. The mean fibrosis stage increased in positive control mice fed a WD SW compared to negative control mice on chow diet (Fig. 4, panels d,e). 4 mice on WD SW and 5 mice on WD SW + BPF99 each had stage 1 fibrosis. The CPA, which was significantly higher in positive controls (vs negative controls), was however reduced in BPF99 treated mice compared to positive controls (WD SW BPF99 vs WD SW vehicle, p < 0.05, Table 2).

**Table 2:** Liver histological findings at the end of the study.

Test	NC NW	WD SW VEHICLE	WD SW BPF 50 mg/Kg/day	P Value	
				NC NW vs WD SW VEHICLE	WD SW Vehicle vs WD SW BPF 99 50 mg/Kg/day
NAFLD activity score	0	21, ± 0,23	1,4 ± 0,16	<0,001	0,054
Steatotis	0	2,9 ± 0,1	2,9 ± 0,1	<0,001	0,99
Lobular inflammation	0	0,8 ± 0,13	0,4 ± 0,16	<0,05	0,08
Hepatocellular ballooning	0	1,3 ± 0,15	1	<0,001	0,07
SAF	0	5 ± 0,29	4,3 ± 0,21	<0,001	<0,05
CPA	1,10 ± 0,15	3 ± 0,3	2,4 ± 0,2	<0,05	0,043

Steatosis, lobular inflammation, cytological ballooning was graded using the NIDDK NASH CRN system. Overall disease activity was assessed using the Steatosis-activity-fibrosis (SAF) system. Fibrosis stage was scored according to the NIDDK NASH CRN system. Fibrosis was also quantified by morphometry and the collagen proportional area (CPA) scored. NC NW (n = 5); WD SW Vehicle (n = 10); WD SW BPF99 (n = 10).

### BPF99 had beneficial effects on several NASH disease-activity related pathways

**Figure 5:**
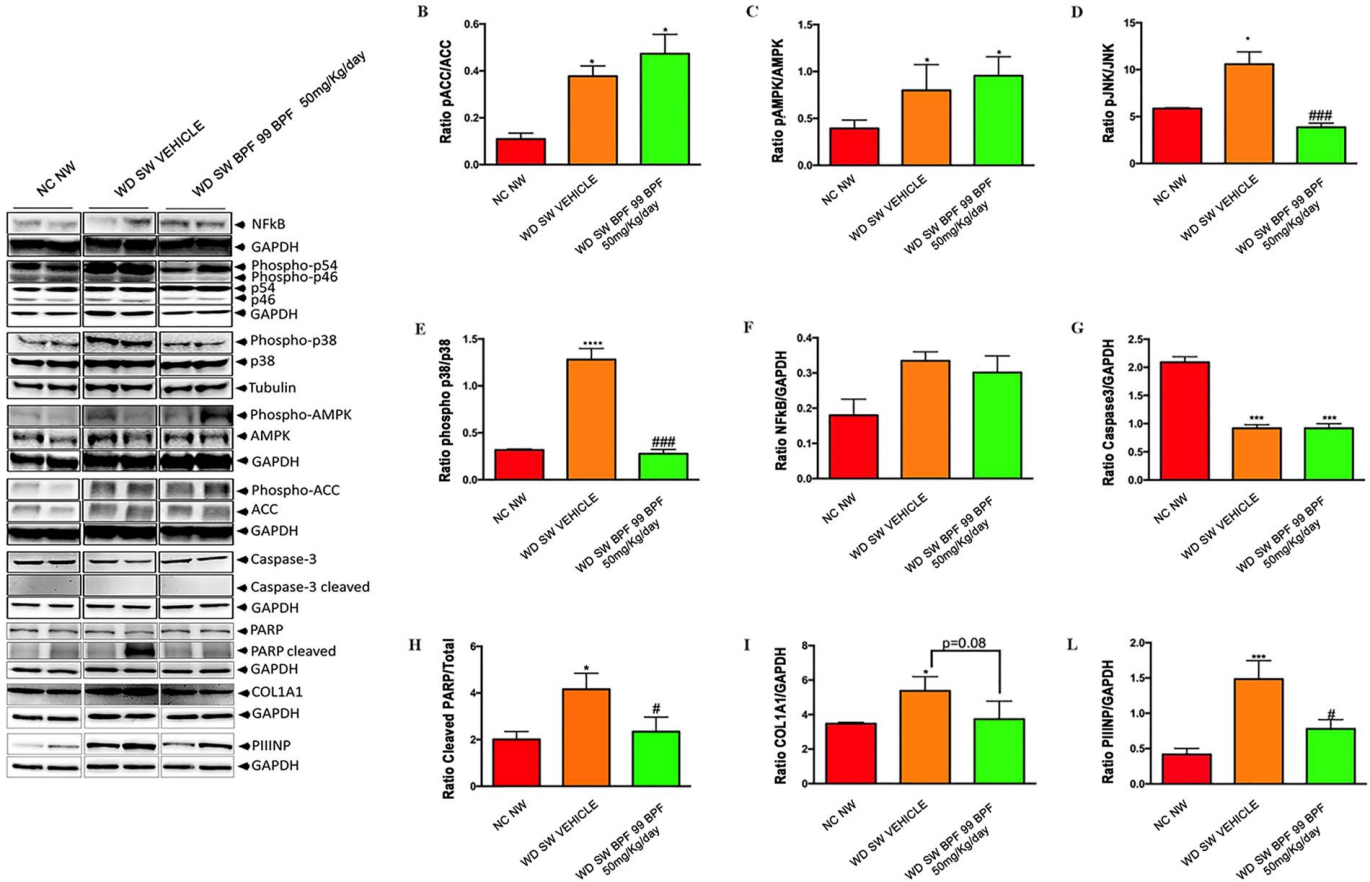
Effects of BPF99 on inflammatory, metabolic and stress-related markers associated with NASH. Whole cell lysates were prepared from liver tissue of mice fed a normal chow diet and tap water (NC NW) or high fructose/glucose, high fat Western Diet, treated with vehicle (WD SW Vehicle) or 50 mg/kg/day BPF99 (WD SW BPF99). (**A**) Immunoblot analysis were performed for NF-kB; phosphorylated and total JNK (p54, p46); phosphorylated and total p38; phosphorylated and total AMPK; phosphorylated and total ACC, Caspase-3; COL1A1; PIIINP; PARP; cleaved Caspase-3 and cleaved PARP product p89 were not visualized despite long exposure times. GAPDH and Tubulin were used as a control for protein loading. Western blot analysis showed that (**B**) pACC/ACC and (**C**) PhosphoAMPK/AMPK ratio levels were up-regulated in WD SW-fed mice treated with vehicle or BPF99. The activation of (**D**) JNK and (**E**) p38 (shown as phosphorylated/total protein ratio) was markedly increased in NASH mice treated with vehicle compared to nonsteatotic controls. Treatment with 50 mg/kg/d BPF99 significantly reduced the phosphorylation levels of the MAP kinases effectors in the livers of WD SW-fed mice compared to the vehicle group compared to NC NW group. (**F**) NF-kB was increased in WD SW-fed mice treated with vehicle or BPF99. (**G**) Caspase-3 levels were similar in NASH animals treated with vehicle or BPF99 and higher compared to control animals. (**H**) PARP protein levels were higher in NASH animals treated with vehicle, whereas BPF99 reduced PARP expression to control levels. (**I**) Procollagen-1 and (**L**) procollagen-3 levels were higher in WD SW-fed mice treated with vehicle compared to control animals. BPF99 was able to reduce Procollagen I and III levels. NC NW (n = 4, red bar); WD SW Vehicle (n = 10, orange bar); WD SW BPF99 (n = 10, green bar). Data are expressed as the mean ± SEM. *p < 0.05, ***p < 0.001 vs NC NW; ^#^p < 0.05, ^###^p < 0.001 vs WD SW Vehicle. Full-length blots are presented in Supplementary Fig. 1A–R.

ACC (total and p-ACC) and AMPK phosphorylation were increased (Fig. 5, panels A, B and C; p < 0.05 for both) in WD SW-fed positive controls compared to chow-fed negative controls. BPF99 did not have a significant effect on either ACC or AMPK, two key metabolic drivers of steatosis and its disposal in NAFLD^40^. In contrast, BPF99 had a highly significant effect on JNK and p38 MAPK phosphorylation returning them to levels seen in chow fed mice and lower than the levels seen in positive controls (Fig. 5, panels A, D and E; p < 0.001 for both). These effects were specific with no impact of BPF99 on NF-kB activation (Fig. 5, panels A and F). BPF99 also decreased PARP (p < 0.05 vs positive controls) which was increased significantly (Fig. 5, panels A and H, p < 0.05) in positive controls vs negative controls.

### BPF99 improved fibrogenic drive

Next, the effect of BPF99 on fibrogenic activity was assessed by evaluating procollagen I expression in the liver of mice fed a chow diet, western diet with sugar water or a western diet with sugar water and concomitant admnistration of BPF99. Procollagen I protein levels were increased in the mice fed a Western diet compared to mice fed a chow diet (Fig. 5, panels A, I; p < 0.05). These were reduced by concomitant administration of BPF99 (p < 0.08 vs western diet with sugar water alone). There was also a statistically significant increase in procollagen III n-terminal peptide (PIIINP) in the liver tissue of mice fed a Western diet with sugar water compared to chow diet-fed mice (p < 0.001, Fig. 5, panels A, L). These increments were abrogated by the concomitant administration of BPF99 (p < 0.05, Fig. 5, panels A, L).

### BPF99 improved oxidative stress status

**Figure 6:**
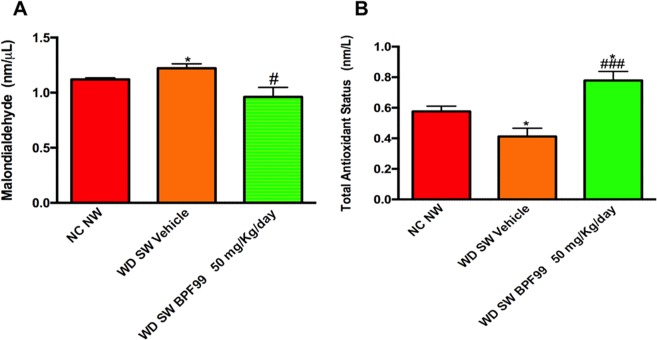
Effects of BPF99 on serum lipid peroxidation and total antioxidant status. Values were measured at the end of the study in serum of NC NW (n = 5, red bar), WD SW Vehicle (n = 10, orange bar) and WD SW BPF99 50 mg/Kg/d (n = 10, green bar) mice. Data are expressed as the mean ± SEM. *p < 0.05 vs NC NW; ^###^p < 0.001 vs WD SW vehicle.

Lipid peroxidation, measured by serum MDA level, was significantly higher in positive control mice fed a WD SW diet compared to negative control mice fed a normal chow diet (p < 0.05, Fig. 6, panel A). MDA levels were significantly attenuated in BPF99 treated mice compared to positive controls (WD SW BPF99 vs WD SW vehicle, p < 0.05, Fig. 6, panel A).

Next, the effect of BPF99 on total antioxidant status was assessed. The mean serum TAS was significantly decreased in mice with steatohepatitis compared to negative control group (Fig. 6, panel B, p < 0.05). BPF99 administration was associated with a significant increase in serum TAS in mice fed a western diet with sugar water (Fig. 6, panel B, p < 0.001 vs western diet with sugar water vehicle). Animals fed a western diet and sugar water, treated with BPF99, exhibited the increases TAS value even in comparison with the mice fed a chow diet (Fig. 6 panel B, p < 0.05 vs negative control).

### BPF99 counteracted oxidative damage in liver

**Figure 7:**
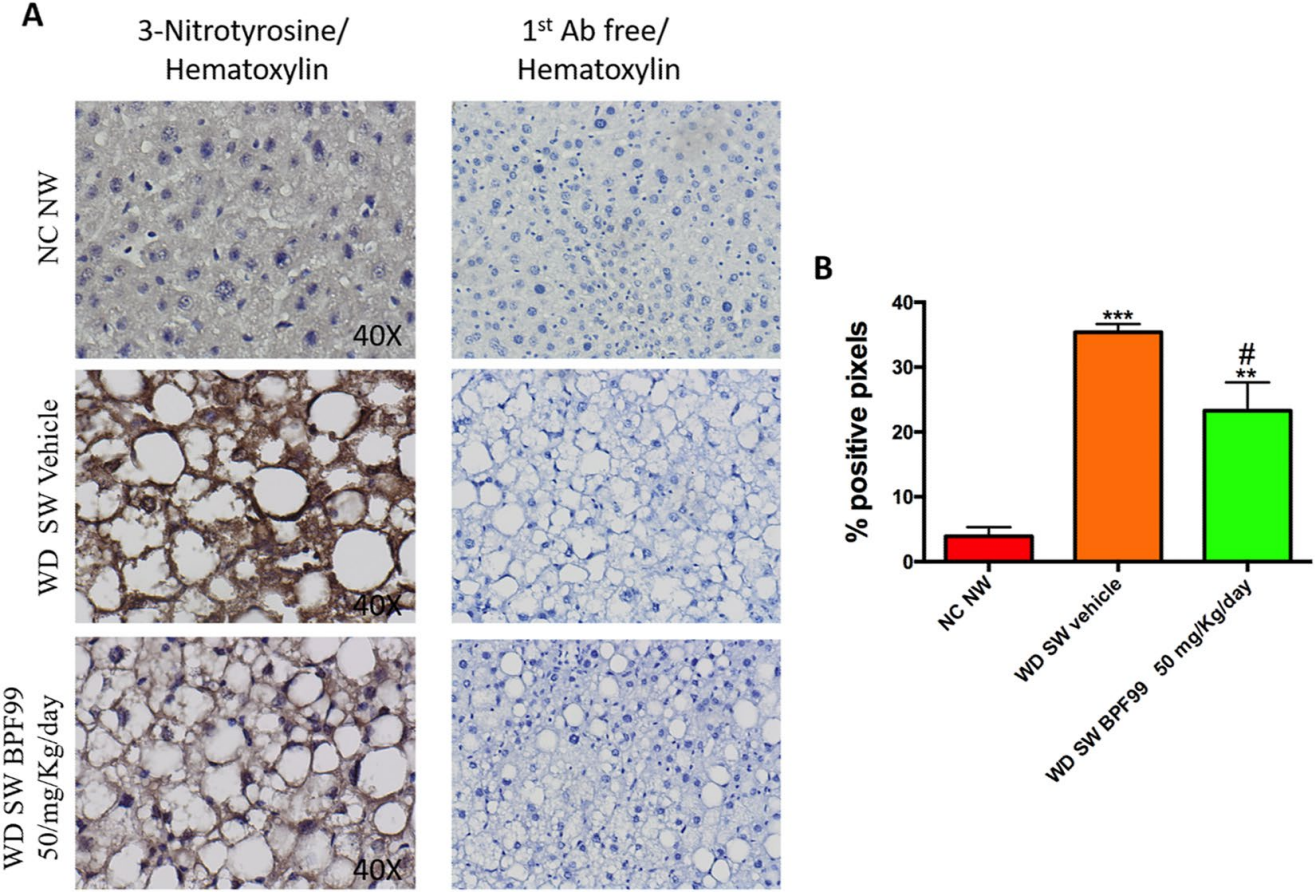
Assessment and quantification of protein nitrotyrosine adducts in the liver. (**A**) Formalin-fixed, paraffin-embedded sections were incubated with antinitrotyrosine antibody followed by anti-rabbit HRP coniugated antibody and 3,3-diaminobenzidine. The nuclei were counterstained with hematoxylin. The degree of staining for 3-NT was significantly higher in the livers of mice fed a WD SW compared to negative control group fed a normal chow diet. A less amount of staining for 3-NT was observed in the livers of mice fed a WD SW and treated with BPF99. Sections without antinitrotyrosine antibody were used as negative control. (**B**) Quantification of differences in peroxynitrite in NC NW (n = 4, red bar), WD SW Vehicle (n = 6, orange bar) and WD SW BPF99 50 mg/Kg/d (n = 6, green bar) was assessed by immunohistochemical staining. DAB-positive pixels were in the 30–115-pixel intensity range in the blue channel of the RGB histogram, whereas background staining was greater than 160 intensity units. The percents of pixels in the 30–115 range in the blue channel are shown. Data are expressed as the mean ± SEM. *p < 0.05, ***p < 0.001 vs NC NW; ^###^p < 0.001 vs WD SW Vehicle.

The degree of staining for 3-NT was significantly higher in the livers of mice fed a WD SW compared to negative control group fed a normal chow diet (p < 0.001, Fig. 7, panel A,B). A less amount of staining for 3-NT was observed in the livers of mice fed a WD SW and treated with BPF99 (p < 0.05 vs WD SW vehicle, Fig. 7 panel A,B), while BPF99 did not restore the degree of staining noted in the negative control group (p < 0.001 vs NC NW, Fig. 7 panel A,B).

## Discussion

NAFLD is the leading cause of liver-related morbidity and mortality. NASH is expected to become the leading etiology of end-stage liver disease and indication for liver transplantation^41^. As drug development efforts are currently focused on NASH and stage 2–3 fibrosis or cirrhosis^4^, the treatment of the greatest burden of disease which characterizes either NAFL or NASH early (stage 0–1) remains an important unmet medical need because the patients still have excess cancer- and cardiometabolic risk^42^.

Thus, the missed opportunity to treat the disease early when the fibrosis is potentially more easily reversible leaves some patients at risk of developing end stage further diseases despite current therapeutic development approaches. There is thus a need for development of simple approaches to enhance diet and life style interventions for those with early stage disease. In the current study, we provide evidence of the potential benefits of BPF99 in a preclinical model of NASH. This model has been validated to develop many of the features of human disease such as obesity, insulin resistance, systemic inflammation with crown-like bodies in adipose tissue, elevated IL-6 and decreased adiponectin^29^. It further sequentially develops steatosis followed by steatohepatitis and then increasing fibrosis which is associated with activation of cellular pathways also activated in human disease. The transcriptomic signature of various phases of the course of NAFLD also resemble that seen in humans. While these studies focused on early stage disease in the mice to generate proof of concept, the similarities between this model and human disease increases the potential for translatability from mice to humans. There are also additional indications that the beneficial effects of BPF99 may extend to more advanced stages of disease as well. This study also provides insights on the potential mechanisms by which BPF99 improves NASH.

Our studies show that neither the body weight nor liver weight decreased with use of BPF99 indicating that decreased weight was not a primary mechanism of action of the compound. In keeping with this lack of effect on body weight, BPF99 did not cause a significant improvement in steatosis grade. On the other hand, it improved insulin sensitivity as measured by the ITT. Thus, even though BPF99 did not reduce overall adiposity, it did improve oxidative damage and peripheral insulin resistance which would be expected to decrease systemic inflammation and thus contribute to the observed improvement in liver histology. At molecular level, this evidence can be attributable to specific intracellular mechanisms, identified primarily in neutralization of free radicals and inhibition of JNK/p38 MAPK pathway which are connected each other, also.

In particular, BPF99 can counteract oxidative stress-induced tissue injury potentiating antioxidant defence exerted reducing peroxynitrite formation.

Moreover, through inhibition of free-radical overproduction, BPF99 can reduce phospho-JNK/p38 MAPK levels characteristic of NASH development. JNK/p38 MAPK activation, subsequent to hepatic saturated fatty acid accumulation, has been associated with the overproduction of pro-inflammatory cytokines^43,44^, the reduction of glucose and insulin tolerance^42,43^, the onset of hepatic insulin resistance^45,46^, and the promotion of gluconeogenesis^47^. Thus, in NASH mice, BPF99-induced modulation of JNK/p38 MAPKs might represent the leading protective mechanism responsible for the amelioration of insulin sensitivity. On the other hand, BPF99-induced protection didn’t depend on any modulation of fatty acids biosynthesis, neither mitochondrial β-oxidation as confirmed by the absence of changes in AMPK-induced ACC phosphorylation.

The inhibitory effect of BPF99 on JNK/p38 MAPKs might be due also to the inhibition of PARP-1 which, in pathological conditions, is over-activated by reactive oxygen and nitrogen species formation. As PARP-1 is considered the direct suppressor of MAPK inhibitor MPK-1, it is plausible that BPF99-induced JNK/p38 MAPK inhibition depends on the reduced PARP-1-mediated downregulation of MPK-1^48^.

Recent studies demonstrated that PARP plays a pivotal role in liver fibrosis and inflammation and that its inhibition reduced fibrosis induced by the bile duct ligation^49^. This finding supported by our results extends previous evidence concerning the role of BPF in the improvement of lipid metabolism. In particular, on the basis of those studies, we can hypothesize that BPF-induced activation of cholesterol 7R-hydroxylase promotes the conversion of liver cholesterol to bile acid, thus favouring their fecal excretion^28^. This effect might prevent PARP-1 activation and, consequently, liver fibrosis as highlighted by reduced expression of collagene types I and III.

This latter result was also supported by histological evidence of decreased perisinusoidal fibrosis which suggests further protective mechanisms induced by BPF99 which involve liver sinusoidal endothelial cells (LSECs).

The main feature of the LSECs is represented by their high endocytic capacity to internalize different molecules and substrates. Among them, oxidized low density lipoproteins (oxLDLs)^50–52^, and procollagen type I and III N-terminal peptides, produced during collagen biosynthesis, represent the inducers of LSEC dysfunction^53^, which represents the most important event preceding fibrosis^54^. The occurrence of oxLDLs internalization depends on the increased levels of plasma lipids and cholesterol promoting systemic inflammation and free radicals formation which are the leading cause of endothelial dysfunction. In this scenario, oxLDLs induce damage and defenestration of liver sinusoidal endothelial cells via LOX1^55^.

Our previous studies demonstrated that, in endothelial cells, LOX-1-mediated accumulation of oxLDLs impaired eNOS activity and promoted NF-kB-mediated iNOS over-expression and peroxynitrite formation^56^. All these effects were antagonized by antioxidants^56^, thus suggesting that BPF99 might prevent LSEC dysfunction counteracting the oxidative damage, highlighted by plasma MDA, and potentiating circulating total antioxidant systems. In accordance with this hypothesis, BPF extracts have shown potent antioxidant properties able to normalize the detrimental effects of altered lipid profile both in humans and in animals. The enhanced levels of plasma total antioxidant systems might be attributable to the main flavonoids in bergamot juice^14^, naringin and hesperidin, which have shown to potentiate the antioxidant status in liver and heart respectively through the upregulation of nuclear factor erythroid 2-related factor 2 (Nrf 2)^57,58^. Moreover, we hypothesize that a further mechanism of BPF99 attributable to naringin and hesperidin is represented by the neutralization of peroxynitrite overproduction detected in the whole hepatic tissue.

Therefore, all these results suggest that, in the early stages of NASH, BPF99 revers the lipid-induced redox-imbalance in mouse liver, probably through the inhibition of ROS/JNK/p38-MAPK pathway, responsible for the overproduction of inflammatory and fibrotic markers.

Altogether the concordance between the decrease in hepatocellular ballooning and inflammation and the key pathways related to oxidative stress-induced cell death and inflammation, other than the reduced fibrogenesis, further support the potential translatability of the findings of this study into humans. In this context, the regression of hepatocellular ballooning is particularly noteworthy since it is a hallmark lesion of NASH^3^.

A key question relates to the active moiety in BPF99 that is responsible for these effects. We have previously shown that BPF99 is composed of a number of biologically active phenols and it is currently unclear if one or more of these contribute to the observed effects. This is potentially relevant for drug development. However, many naturally occurring foods and fruits have biologically active properties that cannot be ascribed to a specific compound. This remains an area for future research. As with many studies, this study also has certain limitations. The study was focused on early stage disease and the effects of BPF99 were not studied in those with more advanced disease. The inability to identify a specific component of BPF99 that drives its effects on NASH may be considered a limitation but should not preclude its use as a food supplement. These data however provide support for development of a regulatory path for such complex mixtures occurring naturally to be developed as a food supplement or drug. In summary BPF99 improved insulin resistance, decreased hepatocellular ballooning, inflammation and fibrosis without a major effect on body weight or steatosis in a diet induced animal model of NAFLD that recapitulates many features of human disease. While much additional work is needed to establish its use in humans for the treatment of NASH, these data provide proof of concept that BPF99 has anti-NASH activity and a rationale to continue its development for the treatment of NASH.

## Supplementary information

Supplementary information
