# Supplementary material for: Bergamot Polyphenols Improve Dyslipidemia and Pathophysiological Features in a Mouse Model of Non-Alcoholic Fatty Liver Disease
Source: Sci Rep. 2020 Feb 13;10:2565. doi: 10.1038/s41598-020-59485-3 (PMC7018973; doi:10.1038/s41598-020-59485-3)
Supplement: Supplementary file 1 — Supplementary information [file 41598_2020_59485_MOESM1_ESM.docx]

**BERGAMOT POLYPHENOLS IMPROVE DYSLIPIDEMIA AND PATHOPHYSIOLOGICAL FEATURES IN A MOUSE MODEL OF NON-ALCOHOLIC FATTY LIVER DISEASE**

Vincenzo Musolino^1,2#;^, PhD; Micaela Gliozzi^1,2^, PhD; Federica Scarano^1,2^; Francesca Bosco^1,2^, PhD; Miriam Scicchitano^1,2^; Saverio Nucera^1,2^; Cristina Carresi^1,2^, PhD; Stefano Ruga^1,2^, Maria Caterina Zito^1,2,^ Jessica Maiuolo^1,2^, PhD; Roberta Macrì^1,2^, PhD, Nicola Amodio^3^, PhD; Giada Juli^3^; Pierfrancesco Tassone^3^, MD; Rocco Mollace^1,2^, MD; Rebecca Caffrey^4^, PhD; Jonathon Marionaux ^4^, MS; Ross Walker^5^, MD; James Ehrlich^6^ MD, Ernesto Palma^1,2^, MD; Carolina Muscoli^1,2^, PhD; Pierre Bedossa^7,8^ MD, PhD, Daniela Salvemini^9^, PhD, Vincenzo Mollace, MD^1,2^; and Arun J. Sanyal, MD^4^

Running title: BPF99 improves NAFLD

# Corresponding author at IRC-FSH Department of Health Sciences, University "Magna Græcia" of Catanzaro, Campus Universitario di Germaneto, 88100 Catanzaro, Italy.

Nutramed S.c.a.r.l.- Complesso Ninì Barbieri, Roccelletta di Borgia, Catanzaro, Italy. Telephone: 00393935674111

[Email: xabaras3@hotmail.com](mailto:xabaras3@hotmail.com)

Senior author: Arun J. Sanyal, MD

Address: 800 E Leigh St, Richmond, VA 23219, United States of America Phone: +1 804-594-6863

[Email: arunjsanyal@gmail.com](mailto:arunjsanyal@gmail.com)

Affiliations:

1. Institute of Research for Food Safety and Health (IRC-FSH), Department of Health Sciences, University "Magna Graecia" of Catanzaro, Italy.
2. Nutramed S.c.a.r.l. Complesso Ninì Barbieri, Roccelletta di Borgia, Catanzaro, Italy.
3. Department of Experimental and Clinical Medicine, University "Magna Graecia" of Catanzaro, Italy.
4. Sanyal biotechnology**,** 800 E Leigh St, Richmond, VA 23219, USA.
5. Macquarie University Medical School, Sydney, Australia.
6. University of Colorado, Denver, CO, USA.
7. Liverpat, Paris, France.
8. Institute of Cellular Medicine, University of Newcastle, UK.
9. Department of Pharmacology and Physiology, Saint Louis University School of Medicine, 1402 South Grand Blvd, St. Louis, MO 63104, USA.

**SUPPLEMENTARY MATERIALS AND METHODS AND SUPPLEMENTARY RESULTS**

***Supplementary materials and methods***

***Extraction and HPLC analysis of flavonoid compounds in serum samples***

*Instrumentation*

The HPLC analysis were performed by RP-HPLC analysis on a Thermo Scientific Ultimate Dionex 3000 UHPLC equipped with an Hypersil gold column (C18) (dim.mm 250 × 4.6, particle size 5 μm), injections loop 20 μl, flow rate 1 ml/min and a mobile phase consisted of the following solvents: A (H20 + TFA 0.1%), B (methanol), as indicated in Table S1.

| Row Gradient | | | |
| --- | --- | --- | --- |
| **Time (min.)** | **Flow (ml/min.)** | **%A** | **%B** |
| **Equilibration** | | | |
| 0 | 1,000 | 95,0 | 5,0 |
| Run | | | |
| 1 | 1,000 | 95,0 | 5,0 |
| 7 | 1,000 | 80,0 | 20,0 |
| 14 | 1,000 | 50,0 | 50,0 |
| 20 | 1,000 | 5,0 | 95,0 |
| 26 | 1,000 | 95,0 | 5,0 |
| 30 | 1,000 | 95,0 | 5,0 |

**Table S1:** HPLC gradient used during the analysis.

Absorbance were obtained at the wavelength of 230 nm, 254 nm and 280 nm. The instrumentation performance, chromatograms, and initial data processing were carried out with Chromeleon software.

*Calibration curve*

The calibration curve was built using flavonoids with high purity (>99%). Naringin, neohesperidin and neoeriocitrin were used as a standard because they are the most abundant flavonoids present in BPF [41]. The compounds were solubilized in H_2_O-MeOH in a ratio 80:20 (Fig. S1 and Fig. S2).


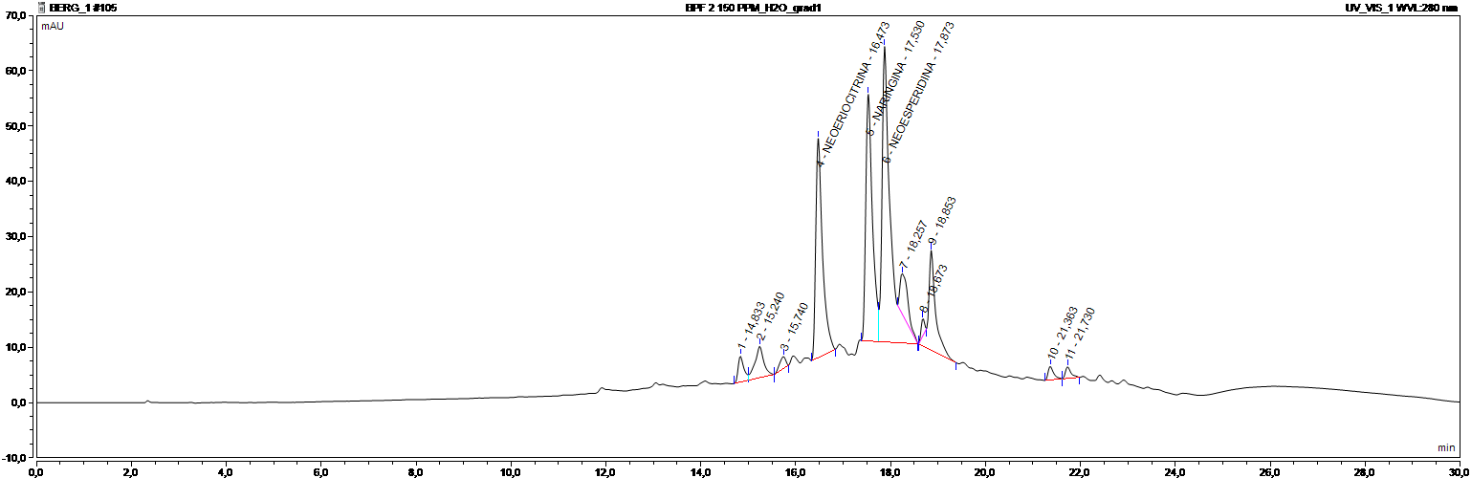


Figure S1: Chromatogram of BPF.


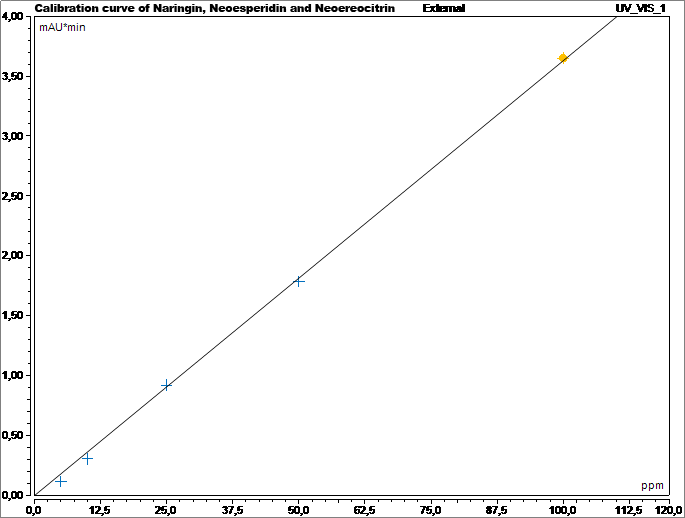


**Figure S2:** HPLC calibration curve.

*Sample preparation*

The serum was collected as described in the “*Blood collection and Analysis of blood based endpoints” section.* Prior to analysis, all frozen subject samples, calibration standards and quality control samples were thawed and allowed to equilibrate at room temperature. Serum samples were acidified using water with TFA (pH 2,5). The flavonoids were then extracted from the serum using a SEP-PAK cartdrige (solid phase extraction). 90/10 MeOH-H_2_O acidified with TFA 0,1% (v/v/v) was used as a conditioning solvent, H_2_O acidified with TFA 0,1% was used as equilibration solvent (v/v), 5% MeOH/H_2_O with 0,1% di TFA (v/v/v) was used as a desalting solution and 50/50 AcCN/H_2_O with 0.1% TFA (v/v/v) was used as elution solvents. The elution solvent (2ml) was evaporated to dryness under nitrogen at 40 ± 2 °C. The dried sample were reconstituted using 0,1 ml of MeOH-H_2_O (80/20 v/v). The total yield of the extraction was more than 90% (data not shown).

***Supplementary results***

Serum concentration of flavonoids after oral administration of BPF99 is illustrated in Table S2. In animals treated with vehicle the main flavonoids of the BPF99 flavonoids were not retrieved in the serum (Table S2, Figure S3). Neoeriocitrin, Naringin and Neohesperidin were found in the serum of the mice treated with BPF99 (Table S2, Fig.S4).

| **Arms** | **Neoeriocitrin (PPM)** | **Naringin (PPM)** | **Neohesperidin (PPM)** |
| --- | --- | --- | --- |
| **NC NW (n=3)** | 0 | 0 | 0 |
| **WD SW Vehicle (n=5)** | 0 | 0 | 0 |
| **WD SW BPF99 (n=10)** | 0,1063 ± 0,001764 | 0,2213 ± 0,003691 | 0,2893 ± 0,01540 |

**Table S2:** Serum concentration of the main three flavonoinds present in BPF.

All three flavonoids were detected, at the end of the experiment, only in the serum on the mice treated with BPF99. Neoeriocitrin was detected at lower concentration compared to naringin and neohesperidin were present.


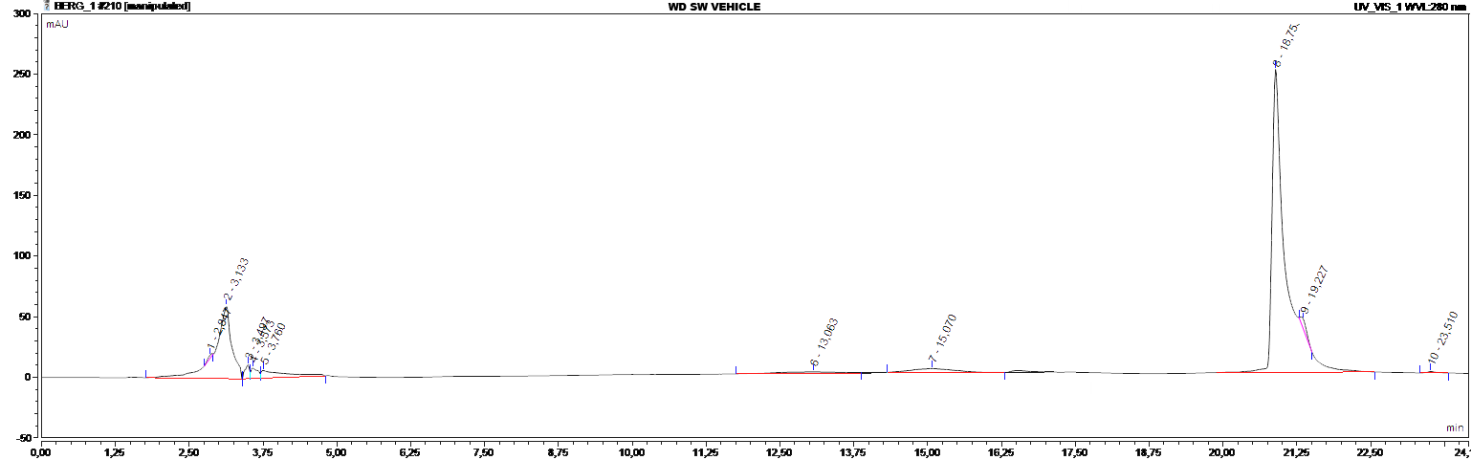


**Fig.S3** Representative chromatogram of WD SW Vehicle serum sample


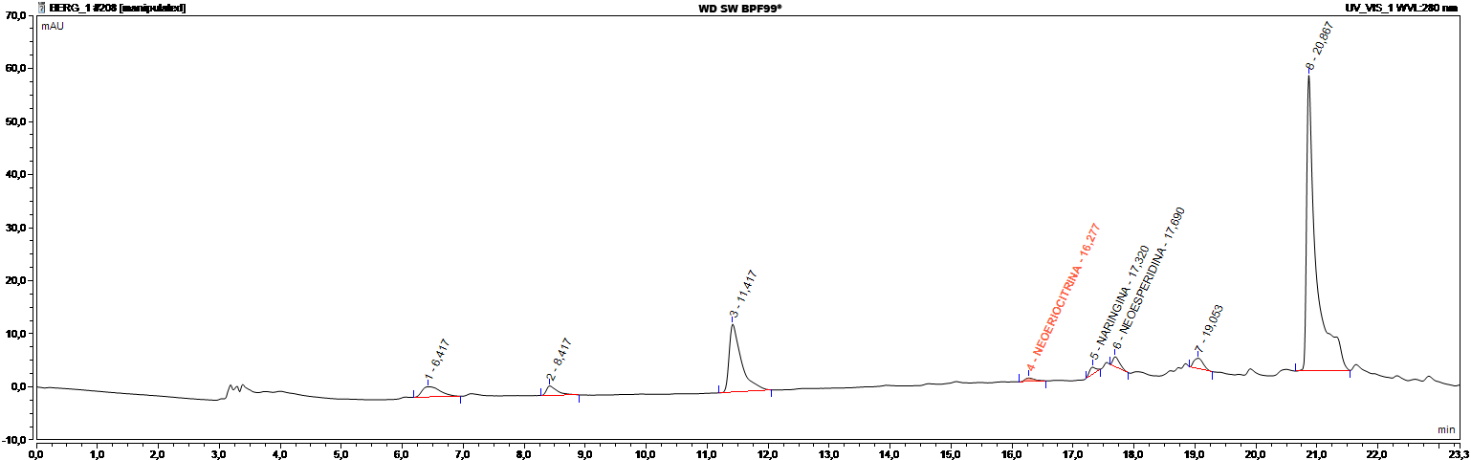


**Fig.S4** Representative chromatogram of WD SW BPF99 serum sample
